# Early hippocampal volume loss as a marker of eventual memory deficits caused by repeated stress

**DOI:** 10.1038/srep29127

**Published:** 2016-07-04

**Authors:** Mohammed Mostafizur Rahman, Charlotte K. Callaghan, Christian M. Kerskens, Sumantra Chattarji, Shane M. O’Mara

**Affiliations:** 1 National Centre for Biological Sciences, Tata Institute of Fundamental Research, Bangalore 560065, India; 2 Institute of Neuroscience, Trinity College Dublin, College Green, Dublin 2, Ireland

## Abstract

Exposure to severe and prolonged stress has detrimental effects on the hippocampus. However, relatively little is known about the gradual changes in hippocampal structure, and its behavioral consequences, over the course of repeated stress. Behavioral analyses during 10 days of chronic stress pointed to a delayed decline in spatial memory, the full impact of which is evident only after the end of stress. In contrast, concurrent volumetric measurements in the same animals revealed significant reduction in hippocampal volumes in stressed animals relative to their unstressed counterparts, as early as the third day of stress. Notably, animals that were behaviorally the worst affected at the end of chronic stress suffered the most pronounced early loss in hippocampal volume. Together, these findings support the view that not only is smaller hippocampal volume linked to stress-induced memory deficits, but it may also act as an early risk factor for the eventual development of cognitive impairments seen in stress-related psychiatric disorders.

The hippocampus, a key component of the episodic and spatial memory system in both humans and rodents, is particularly vulnerable to the effects of severe and prolonged stress. Accumulating evidence from neuroimaging and clinical studies on stress-related psychiatric disorders have reported reduction in hippocampal volume, and this is believed to contribute to memory deficits seen in these debilitating conditions^1^,^2^,^3^,^4^. These findings on abnormalities in hippocampal structure and function are also relevant in light of the central role played by this brain area in the negative-feedback regulation of the stress response via the hypothalamic-pituitary-adrenal (HPA) axis^5^.

A range of animal models have been used to gain mechanistic insights into the impact of repeated stress on the hippocampus at multiple levels of neural organization. For instance, chronic stress causes dendritic atrophy^6^,^7^ and loss of dendritic spines in areas CA1 and CA3^8^, and also reduces the generation of new neurons in the dentate gyrus^9^,^10^. Consistent with these effects, chronic stress reduces brain-derived neurotrophic factor (BDNF), a key regulator of neuronal growth and morphological plasticity^11^,^12^,^13^. Synaptic physiology is also affected –well characterized amongst these effects is the impairment of long-term potentiation (LTP), a synaptic plasticity mechanism thought to be crucial for learning and memory^14^,^15^. Finally, at the behavioral level, stressed rodents also perform poorly on hippocampus-dependent spatial learning tasks^16^,^17^. Together, these detrimental effects of stress at the molecular and cellular levels are believed to impair hippocampal learning and memory^18^,^19^.

As useful as these earlier findings have been in constructing a conceptual framework for animal models of stress, our current understanding is largely based on molecular, cellular and behavioral analyses of stressed versus unstressed animals at fixed time points after the end of stress. Less is known about the gradual and cumulative impact of stress on hippocampal structure and function in the same animal over the course of repeated stress. It is not clear how early or late during the exposure to chronic stress the behavioral and structural effects occur. For instance, do these changes happen in parallel or does one precede the other? To address these gaps in knowledge, we performed a longitudinal study to characterize the temporal progression of stress-induced changes in memory and hippocampal structure in the same animal. To this end, we combined volumetric measurements of the hippocampus using magnetic resonance imaging (MRI) with behavioral assays of hippocampus-based spatial memory tasks at multiple time points during and after a 10-day chronic immobilization stress paradigm (Fig. 1).

## Results

We first confirmed the efficacy of our chronic stress paradigm by assessing its impact on two well-established parameters of stress-induced changes in rodents^13^,^20^,^21^,^22^. First, we compared changes in body weight and found a significant reduction in the percentage weight gained across days in stressed animals compared to unstressed animals (Fig. 2a). Second, we confirmed that this 10-day stress protocol also enhances anxiety-like behavior in the open field test three days after the end of stress (Fig. 2b). The stressed animals spent significantly less time in the center of the arena as compared to the unstressed animals (Fig. 2c (left); *p* = 0.0490). The percentage of path travelled in the center was also significantly lower for stressed animals (Fig. 2c (middle); *p* = 0.0263). However, there was no difference in the total path travelled between the stressed and unstressed rats (Fig. 2c (right); *p* = 0.7024). Thus, the stressed animals showed higher anxiety as evidenced by their avoidance of the most exposed and central part of the arena. Together these two measures confirmed that the chronic immobilization stress paradigm had the same behavioral and physiological impact as reported in earlier studies^13^,^21^.

### Gradual loss of total hippocampal volume over the course of chronic stress

**Figure 1:**
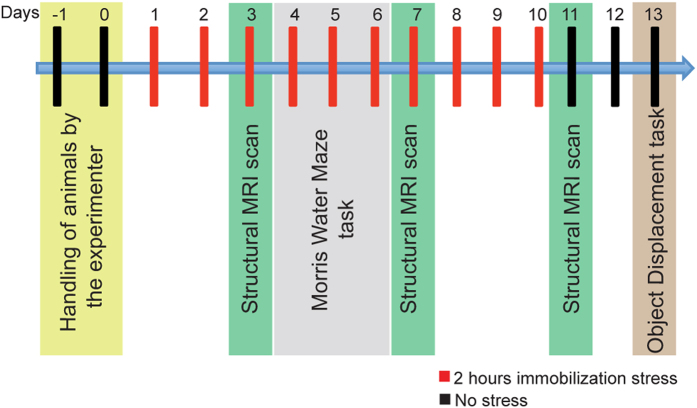
Experimental plan for the study. Each vertical bar represents a single day. The experimental measurement performed on each of the days is indicated below the bars.

We monitored hippocampal volume in the same animal at three different time points during and after the 10-day chronic stress paradigm: during an early stage on day3, a later stage on day7, and after stress on day11 (Figs 1 and 3). Repeated exposure to the same 2-hour immobilization stress over the course of 10 days had a significant effect on total hippocampal volume (Two-way Repeated measures ANOVA: Factor stress: *p* < 0.0001; factor time: *p* = 0.0132; interaction: *p* = 0.1183). Not only was total hippocampal volume significantly lower in stressed animals compared to their unstressed counterparts 1 day after the end of chronic stress (day11), it was significantly lower even during the early (day3) and late (day7) stages of stress (Fig. 3c, *left*). Importantly, this stress-induced reduction took time to build up as the total hippocampal volume on day11 was significantly lower compared to that on day3 in stressed (post hoc Tukey’s multiple comparison test p = 0.0027), but not unstressed, animals (Fig. 3c, *left*). Further, there was no significant effect of chronic stress on the total brain volume (Factor stress: *p* = 0.3857; factor time: *p* = 0.6857; interaction: *p* = 0.9208). Thus, hippocampal volume loss was observed as early as day3 and continued to decrease with the progression of the 10-day chronic stress.

### Asymmetric effects of chronic stress on left versus right hippocampal volumes

A more detailed analysis of the stress-induced reduction in total hippocampal volume revealed that the effects of chronic stress differed between the left and right hippocampi (3-Way Repeated measures ANOVA: Factor stress (between subjects): *p* < 0.001; factor time (within subject): *p* = 0.026; factor hemisphere (within subject): *p* < 0.001; interaction stress*hemisphere: *p* = 0.011; interaction stress*time: *p* = 0.049; interaction hemisphere*time: *p* = 0.830; interaction stress*time*hemisphere: *p* = 0.111). Left hippocampal volumes were smaller compared to those of the right hippocampus across all three days of volumetric measurements. Further, left hippocampal volumes underwent a significant reduction as early as day3 in stressed animals, and this decrease continued to gain in magnitude on day7 and day11 (Fig. 3c, *middle*). In contrast, right hippocampal volumes were significantly lower only one day after the end of chronic stress (day11), and not on days3 and day7 (Fig. 3c, *right*). Further, while the right hippocampal volume was significantly lower on day11 relative to day3 in stressed animals, this was not seen in left hippocampal volumes (Table 1). A significantly greater proportion of the volume loss takes place in the left hippocampus as early as day3 while the full impact of the volume loss is evident only after the end of stress (day11) in the right hippocampus. Taken together, these results show that the same stress affects the left hippocampus much earlier than the right the hippocampus.

### No significant impairment in spatial memory half way through chronic stress

Earlier rodent studies have reported impairments in hippocampus-dependent spatial memory following repeated exposure to stress^15^,^23^. Hence, we examined if similar deficits are evident even halfway through the 10-day chronic stress paradigm used in the present study. To this end, performance of stressed and unstressed animals was tested in a hippocampus-dependent reference memory version of the Morris water maze on days 4, 5 and 6 of the 10-day stress (Figs 1 and 4a,b). This widely used behavioral task evaluates the ability of a rodent to learn to navigate a circular pool using distal cues to locate a hidden, submerged escape platform. During the first session in the maze on day4, both stressed and unstressed rats showed a comparable decrease in escape latencies to reach the platform over the course of the 5 trials (Fig. 4c, *left*). Consistent with these changes in escape latencies, stressed and unstressed animals also showed similar reductions in path lengths taken to escape to the platform. Consequently, this is reflected in similar values for the path length difference within session (i.e. difference between the path lengths on first and last trials within the same session, Fig. 4a) in both groups (Fig. 4d, *left*). Despite achieving similar escape latencies by the end of the 5^th^ trial on the previous day, the escape latency for the stressed group was significantly higher during the first trial of the next session on day5 (Fig. 4c, *middle*; Factor stress: *p* = 0.7936; factor trials: *p* = 0.0012; interaction: *p* = 0.0037; post hoc Fischer’s LSD test). However, despite this initial deficit, stressed animals eventually attained escape latencies similar to those of the unstressed animals by the end of that session (Fig. 4c, *middle*). This contributed to the significantly higher path length difference within session seen in stressed animals compared to unstressed animals on day5 (Factor stress: *p* = 0.0245; factor sessions: *p* = 0.0400; interaction: *p* = 0.1209; post hoc Sidak’s multiple comparison test; Fig. 4d, *middle*). During the third and final session on day6, both stressed and unstressed animals from the very beginning demonstrated reduced levels of escape latencies that were similar to those seen on the last trial of day5 (Fig. 4c, *right*); this caused a significant reduction in path length difference within session on day6 for both groups (Fig. 4d, *right*; post hoc Tukey’s multiple comparison test). The escape latencies did not undergo any further reductions over the course of the remaining trials during the session on day6 (Fig. 4c, *right*; factor stress: *p* = 0.8718; factor trials: *p* = 0.3090; interaction: *p* = 0.8051). Also, escape latencies on day6 were significantly lower than those observed during the first trial on day4 for both stressed and unstressed animals (Fig. 4c, *left and right*; factor stress: *p* = 0.2575; factor days: *p* = 0.0042; interaction: *p* = 0.0794).

Further analysis of changes in path lengths between the three sessions on days 4, 5 and 6 (i.e. difference between path length on last trial of one session and first trial of next session, Fig. 4b) identified a significantly greater difference in stressed animals, pointing to a deficit in retention of spatial memory acquired in the previous session (Fig. 4e; Unpaired t-test *p* = 0.042). We also ruled out thigmotaxis as a potential cause of this difference (Supplementary Figure 1). Despite this retention deficit, the stressed animals were nonetheless capable of eventually acquiring the same level of learning seen in their unstressed counterparts by the end of the last session on day6 (Fig. 4c, *right*). Finally, swimming speed was not affected by stress (Fig. 4f). Together, these observations suggest that there was no significant deficit in the acquisition of spatial memory on the Morris water maze halfway through chronic stress.

### Significant deficit in spatial memory after the end of chronic stress

In view of the absence of any major impairment in spatial memory around the mid-point of the 10 days of chronic stress, we next examined if continued exposure to the same stressor for the full 10 days leads to further behavioral changes. To this end, we subjected both groups to the object displacement task three days after the end of chronic stress (day13, Figs 1 and 5). In this task, which depends on the hippocampus^24^,^25^, spatial memory is measured as a natural exploratory preference of rodents towards a familiar object displaced to a new location while other known objects remain unmoved from their original locations during a previous exposure to the same context. After a 5-minute habituation session in an empty arena (Fig. 5a), rats were exposed to the training context that contained four distinct objects, for four consecutive 3-minute training sessions (Fig. 5a,b; Materials and Methods). During the four training sessions, there was no difference in exploration rate for the four objects or between the two groups (Factor stress: *p* = 0.2097; factor objects: *p* = 0.1204; interaction: *p* = 0.3003). Upon re-exposure to the same context 6 hours later with one of the four familiar objects in a new location (Fig. 5c), the unstressed rats discriminated novel from familiar spatial locations by spending significantly more time exploring the displaced object relative to the three non-displaced objects (Fig. 5d, *left*; factor stress: *p* = 0.0572; factor objects: *p* = 0.0017; interaction: *p* = 0.0417). In contrast, the stressed animals failed to distinguish displaced from non-displaced objects, as there was no difference in the time spent exploring the re-located object during re-exposure to the same context (Fig. 5d, *right*). This impairment in spatial memory was also evident in the greater amount of time spent by the unstressed (*p* = 0.0486), but not the stressed (*p* = 0.8474), animals in the quadrant containing the displaced object (Supplementary Figure 2). Further, no differences in locomotor activity during training or testing were observed between the two groups (data not shown). Together, these results show that 10 days of repeated stress significantly impairs memory for spatial novelty such that stressed animals failed to recognize that a familiar object is in a new location where an object was not encountered earlier.

### Correlations between hippocampal volume loss and impaired performance in hippocampus-mediated spatial memory tasks

A longitudinal study design combining volumetric and behavioral measurements in the same animals enabled us to probe in greater detail the potential links between hippocampal volume and performance in a hippocampus-dependent spatial memory task. First, analysis of all animals (stressed and unstressed) points to a correlation between hippocampal volume and subsequent performance by the same animal in an object displacement task. This correlation also holds for the stressed and unstressed groups separately (Supplementary Table 2). We found a significant correlation between the total hippocampal volume on day11 and performance in the object displacement task (change in exploration rate) on day13 (Pearson’s *r* = 0.59, *p* = 0.008) (Supplementary Figure 3a). Thus, animals that had smaller hippocampal volume were also more impaired in the spatial memory task 2 days later. Having identified a correlation between hippocampal volume and performance in a spatial memory task, we then focused on this same parameter (hippocampal volume on day3) in the stressed group. Remarkably, there was also a significant correlation between the total hippocampal volume on day3 and performance in the object displacement task on day13 (Pearson’s *r* = 0.056, *p* = 0.013) (Supplementary Figure 3b). This suggests that animals with a smaller hippocampal volume as early as the third day of stress ended up being more deficient in the object displacement task after the end of the 10 days of chronic stress. In contrast, the mild impairment seen in the water maze task on day5 exhibited only a weak correlation with the hippocampal volume on day11 (Supplementary Figure 3c). Further, unlike the strong correlation seen with performance in the object displacement task after the end of stress, the total hippocampal volume on day3 showed no significant correlation with the mild impairment seen in the water maze on day5 (Supplementary Figure 3d). Further, correlation analysis of the left and right hippocampal volumes with performance in spatial memory tasks revealed that right, but not left hippocampal volume, on day3 correlated with the performance on object displacement task on day13. However, the both left and right hippocampal volumes on day11 correlated with the performance in object displacement task on day13. These results are summarized in Table 2.

Together these results point to a strong correlation between three parameters – spatial memory after the end of stress (object displacement task on day13) and total hippocampal volume, not only at the end (day11), but also relatively early during chronic stress (day3). To examine this link further, we plotted the population distributions for all animals in the stressed and unstressed groups for all three of these parameters (Fig. 6). For each of the three parameters affected by stress at different time points, the data was fitted with a Gaussian distribution (Materials and Methods; Fig. 6) for the stressed (red dotted lines) and unstressed groups (black dotted lines). Compared to the distribution for unstressed animals (Fig. 6), the distribution for the stressed animals exhibited a shift towards a decrease for all three parameters. For instance, there is a leftward shift (i.e. decrease) in the population distribution for hippocampal volume seen in stressed animals on day3 (Fig. 6). These same animals overlap to a significant degree with the population distribution that underwent a significant downward shift (i.e. decrease) relative to the unstressed distribution of hippocampal volumes on day11. Similarly, this same left-shifted population distribution of hippocampal volume loss on day3 overlaps significantly with the population distribution of stressed animals that showed deficient spatial memory (i.e. downward shift) on day13 (Fig. 6).

The three distributions (on Days 3, 11, and 13) depicted in Fig. 6e are all worse off (i.e. lower volumes and deficient performance) in the stressed animals relative to the unstressed ones. Next,we re-analyzed the same data to understand how these distributions evolve over time, during and after the 10-day stress (Fig. 7). A majority of the animals possessing hippocampal volumes (x-axis of Fig. 7) above the median (i.e. larger hippocampal volumes) are also primarily located above the median along the y-axis (i.e. superior performance in the object displacement task). Conversely, a majority of those that are under the median value for hippocampal volumes are also located below the median value for memory performance. Together these patterns lead to clustering of a clear majority of the animals primarily in the upper right and lower left quadrants on this plot, thereby reiterating the link between hippocampal volume and memory performance (Fig. 6). Moreover, a majority of the points in the upper right quadrant (above the median for both volume and performance) are unstressed animals (black circles), while a majority of those located in the lower left quadrant (below the median for both volume and performance) are stressed animals.How do these volume and behavioral measures evolve over time in the same animal as they are exposed to repeated stress? The arrows (Fig. 7) depict how each animal’s hippocampal volume changes from day3 (black open circle) to day11 (black filled circle) in relationship to their performance in the object discrimination task on day13. The direction of the individual black arrows (connecting black circles) for the unstressed animals is a mixed population – some volumes increase (rightward shift), while others decrease or remain unchanged. In striking contrast, all the red arrows (connecting red circles) show a leftward shift (decrease in volume from day3 to day11). In other words, in addition to the correlations emerging for the population as a whole, *all* the stressed animals are indeed more vulnerable to a continuing decrease in hippocampal volume (leftward arrows), unlike unstressed animals; and all but two of these animals are also more deficient in their memory performance after the end of stress (lower left quadrant).

## Discussion

This study is one of the first attempts to monitor the gradual effects of repeated stress on hippocampal structure and function in the same animal over a period of time. Ten days of chronic immobilization stress had a detrimental effect on hippocampal structure, and its behavioral output. After the end of the chronic stress we found a significant loss in hippocampal volume, as well as impaired spatial memory that depends on the hippocampus. These findings are in agreement with earlier studies that used measurements of stress-induced changes at fixed time points after stress. However, our findings go beyond these earlier reports by showing that the temporal progression of the structural and behavioral deficits follows strikingly different trajectories. At the behavioral level, no significant deficit in spatial memory was observed halfway through the 10-day chronic stress. However, the same animals exhibited considerable impairment in spatial memory after the end of chronic stress. These results point to a delayed manifestation of stress-induced impairment in spatial memory, the full impact of which is only evident after the end of stress. In striking contrast, concurrent hippocampal volumetric measurements in the same animals revealed a relatively early reduction in hippocampal volume after only three days of exposure to stress. Further, the stressed animals that had smaller total hippocampal volumes on the third day of stress were the ones that continued to exhibit significant decrease in volumes a day after the end of stress. And these were also the animals that were prone to bigger deficits in spatial memory after the end of stress. Notably, the smaller hippocampal volume during the early stages of chronic stress exhibited a strong correlation with the eventual decline in spatial memory that emerged after the end of stress.

Previous studies have shown a loss in dendritic branches and spines due to chronic stress^8^,^22^. These results along with reports of impaired neurogenesis^26^ and reduced glial volume and numbers^27^, are consistent with the reduction in volume seen in the present study and earlier reports using models of repeated stress^18^. While earlier studies have reported a loss of hippocampal dendritic spines immediately after stress^28^,such synaptic changes are unlikely to be big enough to contribute significantly to changes in volume. Therefore, future studies will be needed to gain mechanistic insights, including a potential role for impaired neurogenesis and loss of glial cells, into hippocampal volume loss during the early stages of chronic stress.

An interesting aspect of the gradual decrease in hippocampal volume is the asymmetrical effect of chronic stress on the left versus right hippocampus. Initially, a significant loss in volume was evident only in the left hippocampus (day3, Fig. 3c). This reduction in left hippocampal volume continued to gain in magnitude till the end of stress. In contrast, right hippocampal volume showed a significant loss on day11, but not on day3. These results are in agreement with an earlier study in mice reporting a loss only in left hippocampal volume caused by a brief period of stress^29^. An earlier study, using deformation-based morphometry in mice did not detect hippocampal volume loss following the same 10-day chronic immobilization stress^30^. However, it reported a significant increase in ventricular volume after stress. Interestingly, some rodent studies have also suggested asymmetrical effect of stress on the left and right amygdala^31^.

While some studies on patients of stress disorders have reported lower hippocampal volume in one hemisphere only^2^,^32^,^33^, others found hippocampal volume decrease in both hemispheres^23^. It is difficult to draw direct parallels between animal models of stress and clinical studies on stress disorder patients because a complex mix of factors, such as the environmental and genetic causes and history of medications at the time of neuroimaging analyses, are likely to contribute to the mixed results. Despite these caveats, observations on the asymmetric effects of chronic stress are interesting in light of reports on lateralization of several functions in the human brain^34^,^35^. Future studies using animal models will be needed to examine if such asymmetric effects are also evident at the cellular and molecular levels in brain areas affected by stress.

Various rodent models of stress are known to impair performance on hippocampus-mediated spatial memory tasks, such as the Morris water-maze^36^, radial-arm maze^37^, Y-maze^17^, and Barnes maze^38^. Our results on memory deficits in the object displacement task after 10 days of chronic stress add to this growing body of evidence. Interestingly, the same animals did not show any major deficits in spatial memory midway through the 10-day stress paradigm. This suggests that although these animals had already suffered significant loss in hippocampal volume 2 days before they were first tested for spatial learning and memory, this was yet to have a visible impact at the behavioral level (Fig. 8). However, exposure to the same stressor for another 5 days had a far greater impact on both the behavioral and volumetric measures. By day11, i.e. a day after the end of chronic stress, both the left and right hippocampi had undergone even greater volume loss. Moreover, these same animals exhibited a significantly greater impairment when they were tested again for their spatial memory 2 days after the volumetric measurement (Fig. 8). This raises the possibility that the cumulative impact of repeated stress may have to cross a certain threshold of hippocampal volume loss before its detrimental effects are manifested as impaired spatial memory.

There are also certain caveats to the experimental design used in the present study. First, in an effort to restrict the number of times the animals were subjected to the anesthesia followed by MRI imaging, we chose only three time points for volumetric measurements–at an early stage of stress (day3), at a late stage of stress (day7) and after it ended (day11). The unexpected decrease in volume that we detected in stressed animals on day3 highlights the importance of a pre-stress baseline scan. Despite this limitation, the strength of the longitudinal design is evident because the total hippocampal volume on day11 is significantly smaller than that on day3 only in the stressed rats and not in the unstressed animals. Thus, the loss in hippocampal volume is indeed an effect that is specifically due to stress and not just the passage of time. Second, the best way to compare gradual change in spatial memory is to carry out the same memory task across different time points. However, the longitudinal design would require us tosubject the same animals to the same task twice. This would make interpretation of the results difficult as memories formed at an earlier time point would affect the performance at the later time point. Therefore, we needed to use two different tasks that both depend on the hippocampus and provide measures of spatial memory in a similar fashion. Thus, using the object displacement task and the Morris water maze task in the same animals at different time points offered ways around this confound. Importantly, earlier studies have used these two behavioral tasks to report that the extent of deficit in spatial memory in one task is comparable to that seen with the other^25^,^39^,^40^. Another study by Gerstein *et al.*^41^ showed that performance in the Morris water maze task correlates with performance in the Object displacement task, suggesting that they can be used in a comparable fashion to evaluate spatial memory. Despite these earlier reports, it would be useful to further examine if differences in sensitivity of these two behavioral tasks to hippocampal impairment contribute in any way to the emerging deficit at different stages of the chronic stress protocol seen here.

The sequential impact of stress on hippocampal structure and function reported here also points to the need for a better understanding of the physiological mechanisms that co-evolve with stress-induced hippocampal atrophy and memory deficits. A recent study used *in vivo* recordings to examined stress-induced modulation of spatially receptive fields of the hippocampalCA1 ‘place cells’ as mice explored familiar and novel tracks after 5 and 10 days in the same 10-day chronic immobilization stress paradigm used here^42^. In this study, five days of repeated stress caused a reduction in excitability of CA1 pyramidal cells. When the same stress was continued for another 5 days in the same animal, this decreased hippocampal excitability was no longer evident. In other words, while the loss in hippocampal volume continues to gain in magnitude, neuronal excitability exhibits a different trajectory of change over the course of the same 10 days of stress. Two other findings from this study are relevant to our results on hippocampal spatial memory deficits. First, Tomar *et al.* also reported that the hippocampal place cells recorded in a novel context on the 6^th^ day of the 10-day paradigm were largely similar in stressed and control mice. This may explain the absence of a major deficit in the water maze task in the stressed rats in the present study on days 4, 5 and 6. Second, on day11 chronic stress was also shown to limit the ability of the CA1 pyramidal neuronal population to properly distinguish a novel context from a familiar one. Since the object displacement task requires the animal to specifically identify the novel spatial location of a familiar object alongside other non-displaced familiar objects in a familiar context, it is quite likely that deficient context discrimination after 10 days of stress contributes to the impaired performance in this task. Therefore, future studies will be needed to investigate if and how these structural and physiological changes interact and eventually contribute to the buildup in behavioral deficits over the course of chronic stress.

Finally, our finding that the cumulative impact of repeated stress on the structure and behavioral output of the hippocampus is not a homogeneous or linear process, but differs in terms of its development over time, is also relevant to clinical findings. Although a significant body of neuroimaging studies has contributed to the hypothesis that stress produces hippocampal atrophy in PTSD patients, other studies have argued against this interpretation^43^,^44^,^45^. For example, using MRI measurements of hippocampal volume in monozygotic twin pairs, Gilbertson *et al.* concluded that small hippocampal volume represents a pre-existing condition that renders the brain more vulnerable to the development of stress-related psychopathology rather than the neurotoxic effect of the trauma that triggered PTSD^44^. Our results cannot directly address these two views because we did not measure hippocampal volume *before* the animals were exposed to chronic stress. However, the animals having the smaller hippocampal volume soon after the initiation of the stress protocol were the ones that also exhibited a loss in hippocampal volume and poor memory after the end of stress, suggesting that a smaller hippocampus may confer a specific vulnerability to repeated exposure to stress. In this sense, our findings are consistent with the view that hippocampal atrophy may act as an early biomarker of the eventual cognitive deficits seen in patients of stress-related psychiatric disorders^46^. This in turn may help develop prognostic tools to predict susceptibility of an individual to stress-related dysfunction.

## Materials and Methods

### Experimental animals

A total number of 20 adult male Wistar rats (Trinity College Dublin BioResources Unit) weighing 270–300 grams and housed in groups of two were used in the study. They were kept at a 12/12-hour light/dark cycle and had access to water and a standard diet *ad libitum*. All experiments were conducted in accordance with protocols approved by the Animal Ethics Committee of Trinity College Dublin. All experimental procedures (behavioral and imaging) were licensed by the Irish Department of Health and Children and adhered to the relevant European Union (86/609/EEC) and national guidelines.

### Experimental design

The experimental design comprised of experimental procedures conducted over a 15-day period (Fig. 1). The animals were handled on day-1 and day0 to habituate to the experimenter, followed by assignment to stressed (Chronic Immobilization Stress) and unstressed groups randomly on day1. On day3, day7 and day11, the rats underwent a Magnetic Resonance Imaging (MRI) scan to obtain high-resolution structural images of the brain. On day4, day5 and day6 the rats underwent Morris water maze training followed by the object displacement task on day13. To avoid the confound of prior formed spatial memories, two different tasks were used to evaluate spatial memory in the same rat across different time points in the stress paradigm. To this end we used a pair of tasks that have been shown to evaluate spatial memory in a very similar manner^41^. A total of 10 unstressed and 9 stressed rats were used for the analysis in entire study, one stressed rat was left out due to experimental errors.

### Stress Protocol

Rats in the stressed group were subjected to a chronic immobilization stress (CIS) paradigm^22^,^47^, consisting of complete immobilization for 2 hours per day (before noon) in rodent immobilization bags without access to either food or water, for 10 consecutive days. Any experimental procedure was carried out at least one hour after the release from the immobilization bags.

### Body weights

**Figure 2:**
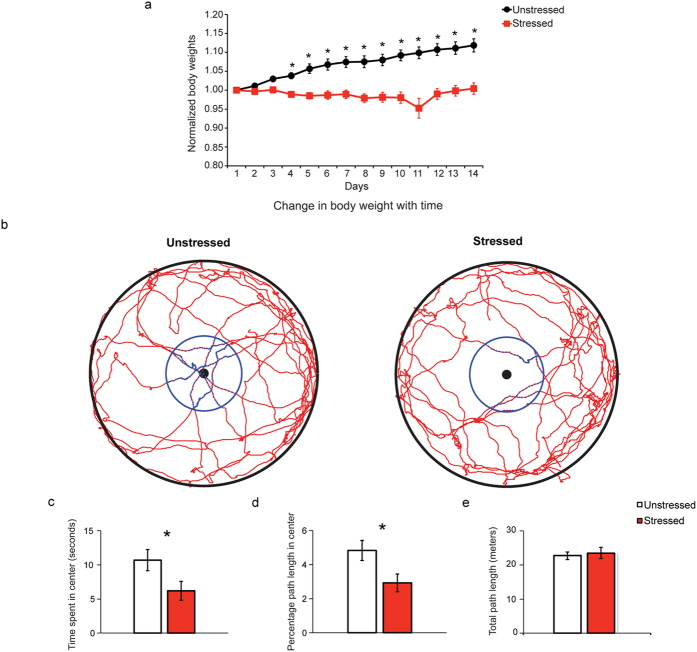
Physiological effects of stress. (**a**) Chronic stress impairs bodyweight gain. Body weights normalized (mean ± SEM) to the body weight on day1 (the day stress commenced) for all the animals. The normalized body weights of the unstressed (N = 10) are significantly greater than the stressed (N = 9) groups from day4 to day14. (Factor stress: *F*_(1, 140)_ = 132.1, *p* < 0.0001; Factor days: *F*_(13, 140)_ = 2.315, *p* = 0.0082; interaction: *F*_(13, 140)_ = 2.872, *p* = 0.0011). Asterisk indicates significant differences (*p < 0.05 level, Sidak’s test for multiple comparisons). (**b**,**c**) Stress enhances anxiety-like behavior measured in the open-field test. (**b**) Representative tracks depicting one unstressed and one stressed animal’s path during open field exploration. The red lines represent the path of the animal. The black dot represents the center of the arena. The black circle represents the outer boundary of the arena. The blue circle represents the boundary of the area defined as the center area of the arena (see materials and methods). (**c**) Quantification of behavior during open-field exploration. The values plotted represent mean. The error bars represent the SEM. **(Left)** Time spent in the center area. **(Middle)** Percentage path length travelled in the center area. **(Right)** Total path length travelled. The stressed (N = 9) group shows significantly lower time (Unpaired t-test: *p* = 0.0490) and percentage path travelled in the center area (Unpaired t-test: *p* = 0.0263) than the unstressed (N = 10) group. An asterisk indicates significant differences (*p < 0.05 level).

Body weights were measured prior to any procedure on each day of the experimental paradigm. The body weights on each day were normalized to the body weight on day1 to obtain the normalized body weights (Fig. 2a).

### Animal preparation for Magnetic Resonance Imaging (MRI)

Animals were anaesthetized with 5% isoflurane (Isoflo, Abbott, Queenboro, England) in oxygen (1 L/min) and maintained with 1.5–2% isoflurane. The level of anesthesia was regularly monitored throughout the procedure using the pedal withdrawal reflex to toe pinch. The animals were placed in a Plexiglas cradle with a three point-fixation system (tooth-bar and ear pieces). Temperature was maintained constant at 37 °C using a warming surface controlled by a water pump-driven temperature regulator. The respiration signal was monitored using custom hardware and software (SA Instruments Inc., Stony Brook, NY, USA). The animals were then placed in a 7 T, 30 cm bore animal MRI system (Biospec 70/30, Bruker Biospin, Ettlingen, Germany) scanner with a circular polarized 1H rat brain RF coil (Bruker, BioSpin). A 7 cm diameter volume coil was used for transmission of the FLASH excitation pulses. Signal detection was performed using a surface coil.

### High resolution anatomical scans

We applied a rapid acquisition with relaxation enhancement (RARE)^48^ sequence for the high resolution anatomical scan (slice thickness: 0.5 mm; number of slices: 54; slice orientation: axial; inter-slice gap: 0.55 mm; TE: 36.7 ms; TR: 5.4595sec; RARE factor: 4; number of averages: 4; FOV: 4 cm × 4 cm; image matrix: 256 × 256; resulting in a spatial resolution of 0.0156 × 0.0156 × 0.05 cm^3^ per voxel and scan duration: 23 min 18 sec). For reproducible anatomical localization of the slice stack, a series of tripilot scans was used to define three mutually orthogonal planes (transversal, horizontal, sagittal). The slice stack was positioned perpendicular to a line connecting the superior end of the olfactory bulb and the superior end of the cerebellum^49^,^50^, with the first slice located at the most posterior point of the cerebellum. In this way, the entire brain was covered (i.e., including olfactory bulb and cerebellum).

### Volumetric analysis of the hippocampus

**Figure 3:**
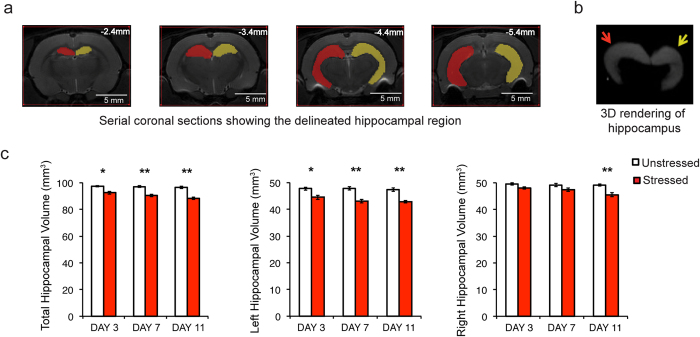
Effects of chronic stress on hippocampal volume. **(a)** Coronal sections showing delineated left (yellow) and right (red) hippocampus. The number on the top-right corner of each image is distance (mm) from bregma. The horizontal white bar in each figure represents the scale-bar. **(b)** A three-dimensional surface rendered reconstruction of the hippocampi. The yellow and red arrows denote the left and right hippocampus respectively. **(c)** Hippocampal volume measurements (mean ± SEM). **(Left)** The total hippocampal volume is significantly lower in the stressed (N = 9) group as compared to the unstressed (N = 10) group on days 3, 7 and 11 (Factor stress: *F*_(1, 17)_ = 58.74, *p* < 0.0001; factor time: *F*_(2, 34)_ = 4.930,*p* = 0.0132; interaction: *F*_(2, 34)_ = 2.274, *p* = 0.1183). **(Middle and right)** The hippocampal volume loss shows an asymmetry between the left and the right hippocampi. The left hippocampal volume is significantly lower in the stressed animals in comparison to their unstressed counterparts on days 3, 7 and 11. However, the right hippocampal volume in stressed animals is significantly lower than the unstressed animals only on day11. There is also a significant reduction in right hippocampal volume of the stressed animals only on day11 in comparison to day3 (Factor stress (between subjects): *F*_(1, 17)_ = 58.738, *p* < 0.001; factor time (within subject): *F*_(2, 34)_ = 4.071, *p* = 0.026; factor hemisphere (within subject): *F*_(1,17)_ = 78.904, *p* < 0.001; interaction stress*hemisphere: *F*_(1, 17)_ = 8.076, *p* = 0.011). An asterisk indicates significant differences (*p < 0.05 level and **p < 0.01, post hoc Tukey’s multiple comparison test).

The scanned images were acquired for signal averaging using ParaVision 4.0 software (Bruker Biospin) for data reconstruction and analysis. The volumetric analysis was done using MIPAV (Medical Image Processing, Analysis, and Visualization) software (Center for Information Technology, National Institute of Health, USA). The identity of the images was encoded to keep the experimenter blind to the group status. Region of interest (ROI) was drawn for the right and left hippocampus and total brain using MIPAV’s paint tools and a standard rat brain atlas^51^(Fig. 3a,b). The method is similar to the one mentioned in previous studies^49^,^50^. The most anterior hippocampal slice included corresponded to a level of approximately −2.04 mm posterior to bregma; the most posterior hippocampal slice included corresponded to a level of approximately −6.96 mm posterior to bregma. Hippocampal structures were identified on nine to ten consecutive slices in the individual animals. For total brain ROI, the most anterior brain slice corresponded to a level of approximately 4.20 mm anterior to bregma and was the first slice in which prefrontal cortex was greater than 50% of brain tissue, and therefore included caudal parts of the olfactory bulb. The most posterior brain slice included was the last slice anterior to the cerebellum. This usually covered the central nucleus of the inferior colliculus and the caudal end of the aqueduct and corresponded to a level of approximately −8.8 mm posterior to bregma. Thus, total brain ROI was distributed over 25–26 consecutive slices in an individual animals. Volumes were calculated using the in-built analysis tool in MIPAV. Hippocampal volumes were also normalized to the total brain volume and expressed as percentage for comparison across time and groups (Supplementary Table 1).

### Morris Water-Maze test

**Figure 4:**
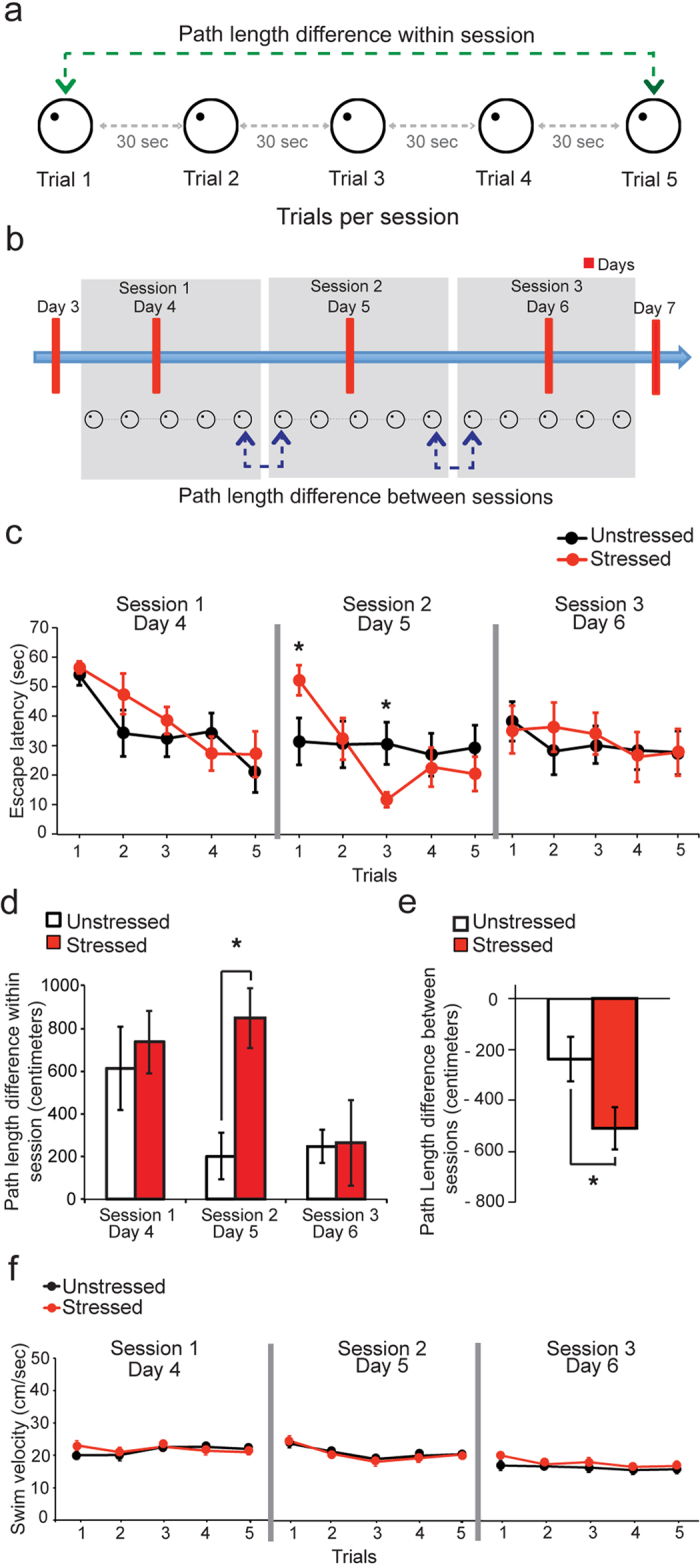
Performance in the Morris Water-Maze task halfway through chronic stress. **(a**,**b)** Protocol for the Morris water maze task. **(a)** Protocol for trials in each session. Each session consisted of five trials with an inter-trial interval of 30 seconds. The difference in the path length of the first and the last trial of a session (green dotted line with arrow) was called the path length difference within a session. **(b)** The same protocol was repeated in session1, session2 and session3 over day4, day5 and day6 respectively. The sum of the differences of the path length in the last trial on day4 and first trial on day5 and the last trial on day5 and the first trial on day6 (blue dotted line with arrow) was called the path length difference between sessions. (**c)** Escape latency in sec (mean ± SEM) for all trials across the three sessions. By the end of the task, both the stressed and unstressed groups of animals acquire spatial memory of the location of the platform. The stressed (N = 9) group shows significantly higher escape latency than unstressed (N = 10) group only during the first trial on session 2 (Factor stress: *F*_(1, 17)_ = 0.07067, *p* = 0.7936; factor trials: *F*_(4, 68)_ = 5.116, *p* = 0.0012; interaction: *F*_(4, 68)_ = 4.289, *p* = 0.0037; post hoc Fischer’s LSD test). **(d)** Path length difference within session for the three sessions (mean ± SEM). It is significantly different between stressed and unstressed group of animals and across sessions (Factor stress: *F*_(1, 17)_ = 6.095, *p* = 0.0245; factor sessions: *F*_(2, 34)_ = 3.542, *p* = 0.0400; interaction: *F*_(2, 34)_ = 2.249, *p* = 0.1209). The stressed (N = 9) group shows significantly higher path length difference within session than the unstressed (N = 10) group in session2 (post hoc Sidak’s multiple comparison test). (**e)** Path length difference between sessions (mean ± SEM). The stressed (N = 9) group has a significantly more negative path length difference between sessions as compared to the unstressed (N = 10) group (Unpaired t-test: *p = *0.042). **(f)** Swim velocity for all the trials across the three sessions (mean ± SEM) do not vary for the stressed and unstressed animals. **(c–e)** An asterisk indicates significant differences (*p < 0.05 level).

The Morris water-maze consisted of a black circular pool filled with water at 25 degree Celsius (diameter 150 cm, height 35 cm), situated in a room with two extra-maze cues. On training days, rats were trained to find a platform (diameter 9.5 cm, height 29 cm) hidden 2 cm below the water surface^52^,^53^, at a fixed location in one of the maze’s quadrants. The rat was introduced into a randomly chosen quadrant and allowed to find the platform. It was guided to the platform after 60 sec, if it failed to find the hidden platform in any single trial. Rats were given five trials/day for 3 days with a 30 sec inter-trial interval (ITI) (Fig. 4a). The same protocol was repeated on day4, day5 and day6 as session1, session2 and session3 respectively (Fig. 4b). Swim paths were recorded and analyzed with an image analysis system (EthoVision, Noldus, Wageningen, Netherlands) to obtain the escape latency (time taken to find the platform), path length (distance moved before reaching the platform) and swim velocity for each trial. Path length difference within session was used to assess the acquisition of spatial memory in each session. This was calculated by subtracting the path length of trial 5 from the path length of trial 1 in each session (green dotted line and arrow; Fig. 4a). Path length difference between sessions was used to evaluate spatial memory retention across sessions. This was calculated by subtracting the path length in trial 1 of session2 from the path length in trial 5 of session1 and subtracting the path length in trial 1 of session3 from the path length in trial 5 of session2 (blue dotted line and arrow; Fig. 4b). The difference of path length between session1 and session2 and between session2 and session3 were summed up for each individual and used for further analysis.

### Object displacement test

**Figure 5:**
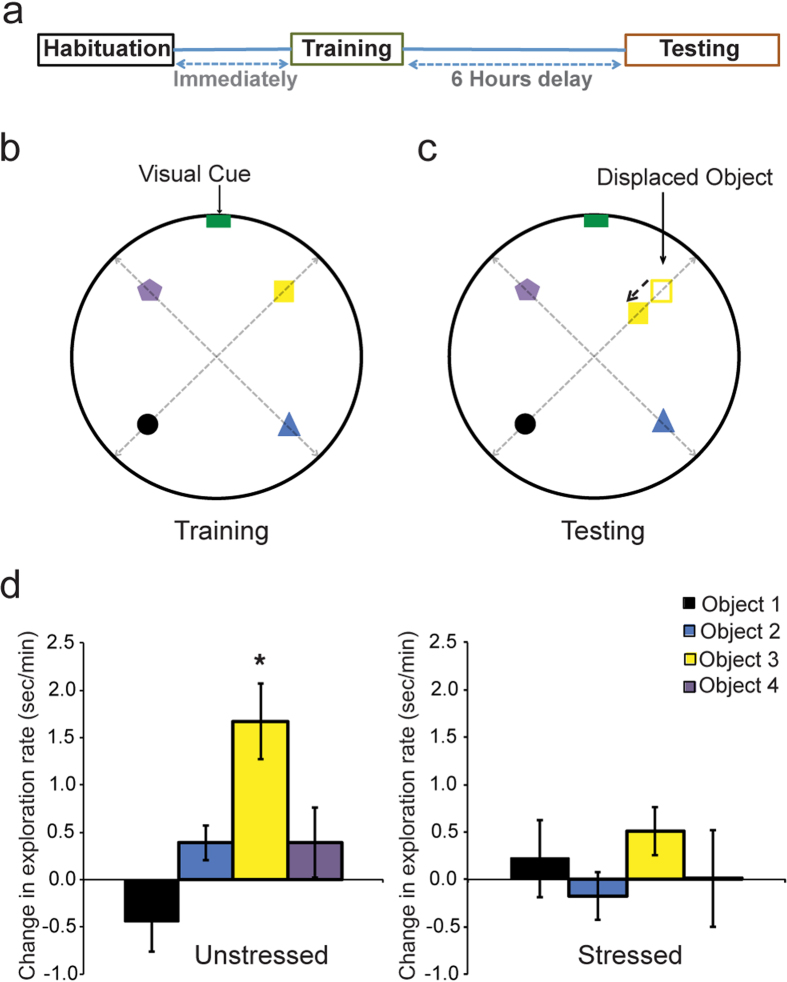
Performance on the object displacement task after the end of chronic stress. **(a)** Protocol: The habituation phase was followed immediately by training phase. The testing phase was conducted 6 hours after the end of training (see materials and methods). (**b)** The location of the four objects in the arena along with the visual cue during the training sessions. (**c)** The location of the four objects in the arena along with the visual cue during the testing session. The object represented by the yellow square is the displaced object. The empty yellow square represents the location in the training session. The black arrow represents the direction of the displacement in location. **(d)** The change in exploration rate of each object for the unstressed (N = 10) and stressed (N = 9) group (mean ± SEM). The yellow bars represent object3 (displaced object). The unstressed group shows a significant increase in exploration rate of displaced object, i.e. object3 as compared to the other objects. The stressed group does not show any significant difference in the change of exploration for all the four objects (Factor stress: *F*_(1, 17)_ = 4.160, *p* = 0.0572; factor objects: *F*_(3, 51)_ = 5.838, *p* = 0.0017; interaction: *F*_(3, 51)_ = 2.942, *p* = 0.0417). An asterisk indicates significant differences (*p < 0.05 level, Tukey’s test for multiple comparisons).

Spatial memory was assessed using an object displacement task^53^,^54^. The apparatus consisted of a black, circular cardboard arena (diameter = 90 cm, height = 35 cm) surrounded by black curtains with one extra-maze cue (white and black stripe pattern of dimension 10 cm × 6 cm) attached and four objects of similar dimension and weight viz., a brown glass bottle (object1), a metal jug (object2), a porcelain vase (object3) and a transparent glass bottle (object4). The test consisted of three phases (Fig. 5a). Initially, the animals were allowed to habituate in the open arena for five minutes, followed by training phase consisting of four sessions of three minutes each, with an inter-session interval (ISI) of five minutes, during which the rats were kept in the home-cage in a separate room. In the object identification training sessions, four objects were placed in a square formation, approximately 30 cm from the center of the arena (Fig. 5b). In the testing session, which consisted of a single session for five minutes, one of the objects (porcelain vase) was displaced by 10 cm towards the center (Fig. 5c). After every session the arena and objects were wiped with alcohol to remove odor traces. The behavior was recorded with a ceiling-mounted camera for all sessions. Object exploration was evaluated by the time spent in contact with the object. Total exploration time for each object was normalized to the total session time to obtain the ‘exploration rate’ for each object. The difference in exploration rate between testing and training phase was used to evaluate the animal’s response to the objects. The movement of the animals was also tracked throughout the task using MouseLabTracker software. The difference of time spent in each quadrant during testing and training sessions was also quantified to evaluate the performance in the task.

### Open field exploration

The movement of the rats was tracked during the habituation phase in the object displacement task, which resembled an open field exploration task^55^, using MouseLabTracker software^56^. The time spent and the path travelled in the center region, defined as a circular region with the center being the center of the arena and radius being one-third the radius of the arena (15 centimeters), was calculated for further analysis (Fig. 2b).

### Correlation Analysis

**Figure 6:**
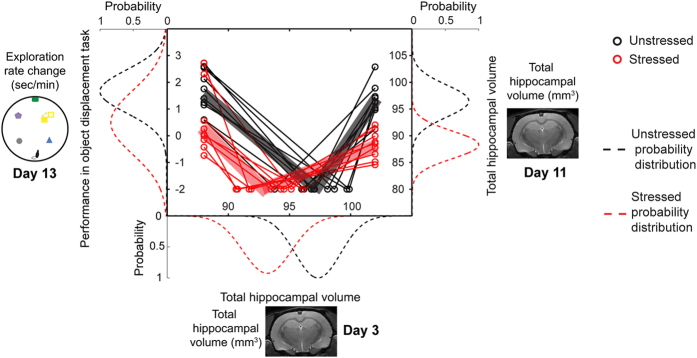
Summary of the effect of stress on hippocampal volume and its correlation with spatial memory deficit. Each circle and line represents a single animal. The dotted lines denote the probability distribution of the stressed (red) and unstressed (black) group of rats. Stress causes a loss in hippocampal volume on day3 and day11 (indicated by the shift of the probability distribution of stressed animals in the bottom and right axes). Stress also causes an impaired performance in spatial memory task (indicated by the shift of the probability distribution of stressed animals in the left axis). Animals having lower hippocampal volume on day3 have lower hippocampal volume on day11; these animals perform more poorly in the ODT on day13. Light red and light black lines depict the average trend for the stressed and unstressed animals respectively.

**Figure 7:**
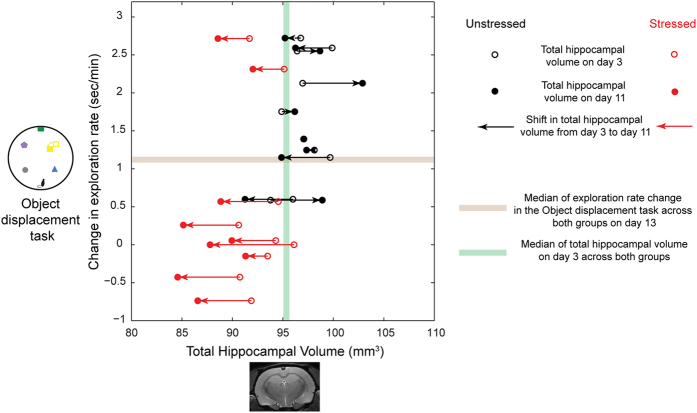
Distribution of memory performance and hippocampal volumes for individual animals and their evolution over time. Each pair of circles connected by a line represents a single animal (black and red represent unstressed and stressed animals respectively). The y-axis represents the performance in the Object displacement task on day13 and x-axis represents the total hippocampal volume. The hollow circle represents the hippocampal volume on day3 and the solid circle the hippocampal volume on day11. The arrowhead shows the direction of the shift in total hippocampal volume from day3 to day11 for each individual animal. The brown line represents the median (50 percentile) of performance in Object displacement task on day13. The green line represents the median (50 percentile) of the total hippocampal volume on day3. A majority of the animals possessing hippocampal volumes (x-axis) above the median (i.e. larger hippocampal volumes) are also primarily located above the median along the y-axis (i.e. superior performance in the object displacement task). Conversely, a majority of those that are under the median value for hippocampal volumes are also located below the median value for memory performance. Moreover, a majority of the points in the upper right quadrant (above the median for both volume and performance) are unstressed animals (black circles), while a majority of those located in the lower left quadrant (below the median for both volume and performance) are stressed animals. The direction of the individual black arrows (connecting black circles) for the unstressed animals is a mixed population – some volumes increase (rightward shift), while others decrease or remain unchanged. In striking contrast, all the red arrows (connecting red circles) show a leftward shift (decrease in volume from day3 to day11).

**Figure 8:**
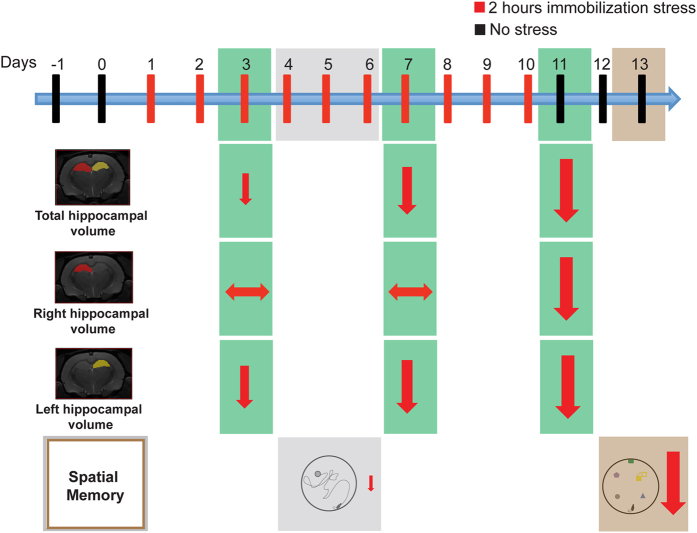
Summary of the different temporal development of structural and behavioral deficits caused by chronic stress. Stress causes a loss in total hippocampal volume as early as day3, which progresses with the 10-day chronic stress. The right hippocampal volume loss is evident only after chronic stress, but the left hippocampal volume loss is evident as early as day3 and progresses with the 10-day chronic stress paradigm. The spatial memory impairment caused by stress also varies over time. A trend of deficit in spatial memory was observed on day5 in Morris water maze task, however the same animals show a strong deficit in spatial memory on day13 observed in object displacement task.

A Pearson correlation coefficient was calculated between the total hippocampal volume (on day3 and day11) and the performance in spatial memory tasks (Morris water-maze task and Object displacement task) (Fig. 6a–d). These total hippocampal volume and performance in spatial memory tasks measurements were used to summarize the results in Fig. 6c. For each of the parameters the values of individual animals were plotted using circles connected by solid lines. These values were used to generate a probability distribution (red and black dotted lines). The probability distribution was generated by boot sampling the values and then fitting the obtained distribution to a Gaussian distribution. Thus, the unstressed and stressed groups have different probability distributions. The shift in direction and distance of the probability distribution of the stressed group indicated the effect of stress.

### Statistical Analysis

Unless otherwise stated repeated measures analysis of variance (ANOVA) was used to compare the hippocampal volume, Morris water maze task, Object displacement task and body weight data across time and groups. Post hoc Sidak’s test was used to compare across groups and Tukey’s test was used to compare within groups. Unpaired t-test was used to compare the data in the open field exploration task.

All statistical analysis was performed using GraphPad prism (GraphPad software Inc., Avenuda de la Playa, La Jolla, CA, USA), except the three-way ANOVA, which was, carried out using SPSS (IBM Corporation, Armonk, New York, USA). All results are presented as mean ± SEM unless otherwise stated. For correlation analysis Pearson’s r was calculated along with the p-value, testing the null hypothesis that there is no correlation between the two quantities. The criterion for statistical significance was p < 0.05.

## Additional Information

**How to cite this article**: Rahman, M. M. *et al.* Early hippocampal volume loss as a marker of eventual memory deficits caused by repeated stress. *Sci. Rep.* **6**, 29127; doi: 10.1038/srep29127 (2016).

## Supplementary Material

Supplementary Information

## Figures and Tables

**Table 1:** Volumetric measures in unstressed and stressed animals.

Measure	Unstressed (N = 10)	Stressed (N = 9)	Unstressed *vs* stressed (difference)
Day 3 TBV (mm^3^)	1352.72 ± 8.36	1341.79 ± 10.36	ns
Day 7 TBV (mm^3^)	1354.33 ± 8.83	1348.64 ± 9.05	ns
Day 11 TBV (mm^3^)	1350.66 ± 8.15	1340.31 ± 10.38	ns
Day 3 THV (mm^3^)	97.31 ± 0.58	92.50 ± 1.06	**
Day 7 THV (mm^3^)	96.99 ± 0.74	90.41 ± 1.06	****
Day 11 THV (mm^3^)	96.51 ± 0.96	88.31 ± 0.86	****
Day 3 LHV (mm^3^)	47.71 ± 0.54	44.48 ± 0.80	**
Day 7 LHV (mm^3^)	47.84 ± 0.61	43.03 ± 0.69	****
Day 11 LHV (mm^3^)	47.38 ± 0.73	42.84 ± 0.43	****
Day 3 RHV (mm^3^)	49.60 ± 0.46	48.02 ± 0.37	ns
Day 7 RHV (mm^3^)	49.16 ± 0.62	47.38 ± 0.61	ns
Day 11 RHV (mm^3^)	49.13 ± 0.38	45.48 ± 0.80	****

Stress causes a loss in total hippocampal volume (Factor stress: *F*_(1, 17)_ = 58.74, *p* < 0.0001; factor time: *F*_(2, 34)_ = 4.930,*p* = 0.0132; interaction: *F*_(2, 34)_ = 2.274, *p* = 0.1183). Interestingly, the stress induced loss in hippocampal volume was asymmetrical across both the hemispheres (Factor stress (between subjects): *F*_(1, 17)_ = 58.738, *p* < 0.001; factor time (within subject): *F*_(2, 34)_ = 4.071, *p* = 0.026; factor hemisphere (within subject): *F*_(1, 17)_ = 78.904, *p* < 0.001;interaction stress*hemisphere: *F*_(1, 17)_ = 8.076, *p* = 0.011).TBV is Total Brain Volume, THV is Total Hippocampal Volume, LHV is Left Hippocampal Volume, and RHV is Right Hippocampal. The day indicated is the day in the experimental paradigm mentioned in Fig. 1. (ns denotes not significant, *p < 0.05, **p < 0.01, ***p < 0.001, ****p < 0.0001).

**Table 2:** Correlation analysis for volumetric measurements and behavioral tasks.

Volumetric Measure/ Behavioral task	Object exploration on Day 13	Morris Water-maze (session 2) on Day 5
Day 3 THV	0.5603 (*)	−0.2820 (ns)
Day 3 LHV	0.2513 (ns)	−0.2470 (ns)
Day 3 RHV	0.4625 (*)	−0.2307 (ns)
Day 3 Left Hippocampal dominance	−0.0121 (ns)	−0.1434 (ns)
Day 11 THV	0.5871 (**)	−0.4827 (*)
Day 11 LHV	0.5024 (*)	−0.5827 (**)
Day 11 RHV	0.5581 (*)	−0.2657 (ns)
Day 11 Left Hippocampal dominance	0.0426 (ns)	−0.4338 (ns)

ns: not significant, *p < 0.05, **p < 0.01. THV is Total Hippocampal Volume, LHV is Left Hippocampal Volume and RHV is Right Hippocampal Volume. Left Hippocampal dominance is the calculated as (LHV-RHV)/THV. The day indicates the day in the experimental paradigm mentioned in Fig. 1. The values are the Pearson’s r calculated for the pair of volumetric measurement and behavioral task.
