# Supplementary material for: Early hippocampal volume loss as a marker of eventual memory deficits caused by repeated stress
Source: Sci Rep. 2016 Jul 4;6:29127. doi: 10.1038/srep29127 (PMC4931588; doi:10.1038/srep29127)
Supplement: Supplementary Information [file srep29127-s1.pdf]

Early hippocampal volume loss as a marker of eventual memory deficits caused by repeated stress

Running Title: Gradual impact of chronic stress on the hippocampus

Mohammed Mostafizur Rahman[1,2], Charlotte K. Callaghan[2], Christian M. Kerskens[2], Sumantra Chattarji[1*], Shane M. O'Mara[2*]

[1]National Centre for Biological Sciences, Tata Institute of Fundamental Research, Bangalore 560065, India
[2]Institute of Neuroscience, Trinity College Dublin, College Green, Dublin (IRELAND), Zip code: D2

* **Corresponding Authors**: *Sumantra Chattarji*
National Centre for Biological Sciences
GKVK Campus,
Bangalore-560065
INDIA
Tel: +91-80-2366120
E-mail: shona@ncbs.res.in

Shane M. O'Mara
Lloyd Building,
Trinity College Institute of Neuroscience,
Trinity College Dublin,
Dublin 2,IRELAND
Tel: +353-01-896-8447
E-mail: shane.omara@gmail.com

Supplementary Table 1

Measure	Unstressed (N=10)	Stressed (N=9)	Unstressed *vs* stressed (difference)
Day 3 THV/TBV (%)	7.20±0.06	6.89±0.06	*
Day 7 THV/TBV (%)	7.16±0.07	6.71±0.08	***
Day11 THV/TBV (%)	7.15±0.08	6.59±0.09	****
Day 3 LHV/TBV (%)	3.53±0.03	3.31±0.05	**
Day 7 LHV/TBV (%)	3.53±0.05	3.19±0.05	****
Day 11 LHV/TBV (%)	3.51±0.06	3.20±0.05	***
Day 3 RHV/TBV (%)	3.67±0.05	3.58±0.03	ns
Day 7 RHV/TBV (%)	3.63±0.05	3.51±0.05	ns
Day11 RHV/TBV (%)	3.64±0.04	3.39±0.06	**

Hippocampal volumetric measurements in unstressed and stressed animals expressed as a percentage of total brain volume. Stress causes a loss in total hippocampal volume (Factor stress: $F_{(1, 17)}$ =40.20, p<0.0001; factor time: $F_{(2, 34)}$ =3.381, p=0.0458; interaction: $F_{(2, 34)}$ =1.767, p=0.1862). Interestingly, the stress induced loss in hippocampal volume was asymmetrical across both the hemispheres (Factor stress (between subjects): $F_{(1, 17)}$=40.197, p<0.001; factor time (within subject): $F_{(2, 34)}$=3.381, p=0.046; factor hemisphere (within subject): $F_{(1, 17)}$=61.845, p<0.001; interaction stress*hemisphere: $F_{(1, 17)}$=8.016, p=0.012). TBV is Total Brain Volume, THV is Total Hippocampal Volume, LHV is Left Hippocampal Volume, and RHV is Right Hippocampal. The day indicated is the day in the experimental paradigm mentioned in **Figure1**. (ns denotes not significant, * denotes p<0.05, ** denotes p<0.01, *** denotes p<0.001, **** denotes p<0.0001).

Supplementary Table 2

Volumetric Measure/ Behavioral task	Object exploration on Day 13	Morris Water-maze (session 2) on Day 5
Day 3 THV Unstressed	0.4103 (**)	-0.0739 (ns)
Day 3 THV Stressed	0.2040 (*)	0.0189 (ns)
Day 11 THV Unstressed	0.2146 (*)	-0.4013 (*)
Day 11 THV Stressed	0.5229 (**)	0.1780 (ns)

Correlation analysis for volumetric measurements and behavioral tasks for each group (stressed and unstressed). ns: not significant, *: $p<0.05$, **: $p<0.01$. THV is Total Hippocampal Volume. The day indicates the day in the experimental paradigm mentioned in **Figure1.** The values are the Pearson's r calculated for the pair of volumetric measurement and behavioral task. The Pearson's r was calculated for the stressed and unstressed group of rats separately. Bootstrapping method was used to gain significant confidence interval.

Supplementary Figure1

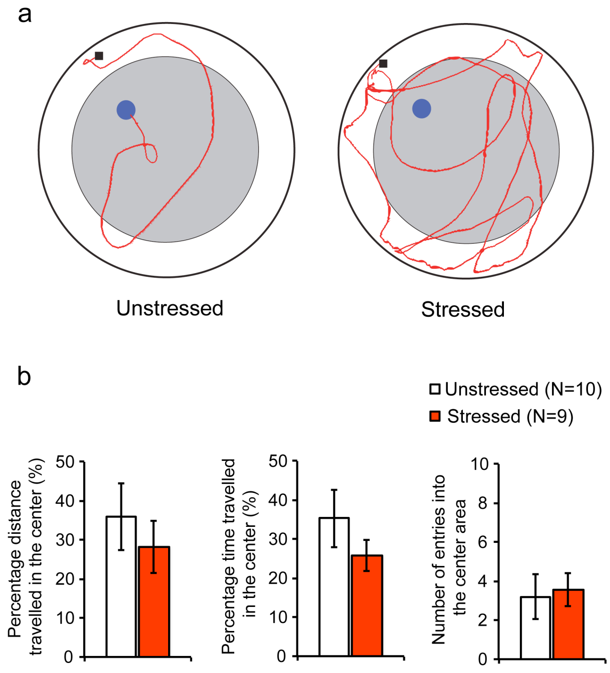

No stress induced thigmotaxis-like behavior during Morris water maze task. (a) Tracks of one representative animal each from the stressed and unstressed group of rats. The black circle represents the boundary of the water maze. The grey region in the center is the defined center area. The purple circle represents the submerged platform. The red line denotes the track for the animal and the black square represents the starting point of the animal.**(b)** The stressed group of rats does not spend more time in the periphery of the maze. The left figure shows no significant difference in the percentage path travelled in the center area between the stressed and unstressed groups (students t-test p=0.489). The middle figure shows no significant difference in the percentage time spent in the center area between the stressed and unstressed groups (students t-test p=0.293). The right figure shows no significant difference in the number of entries to the center area between the stressed and unstressed groups (students t-test p=0.811).

Supplementary Figure 2

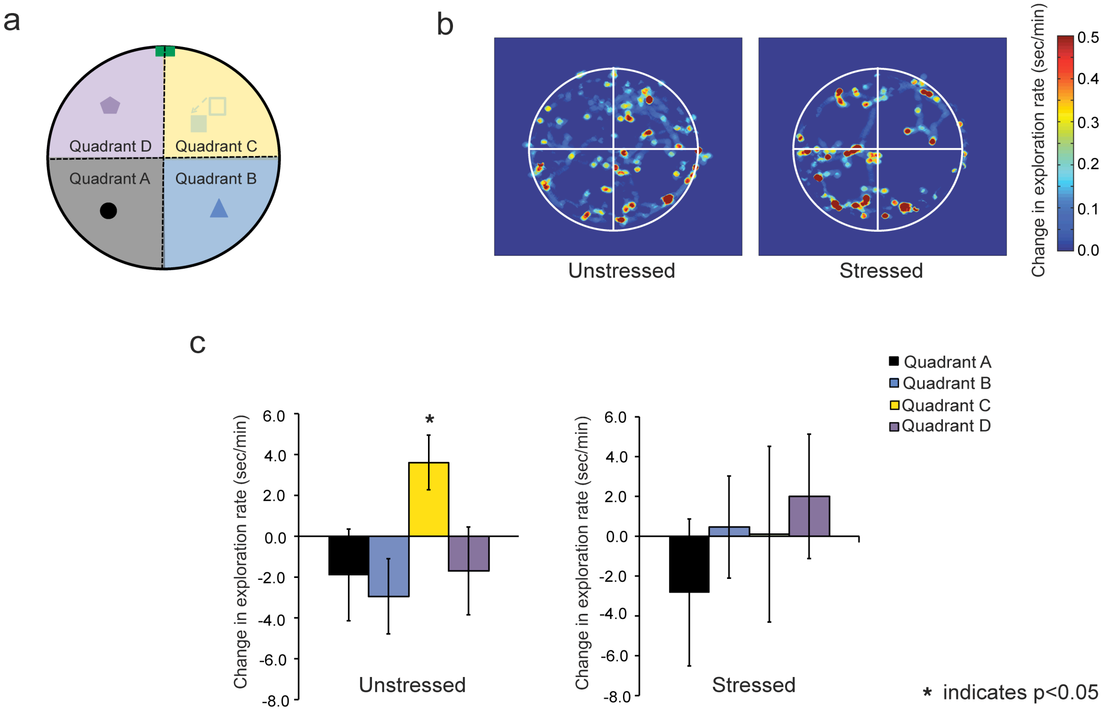

Chronic stress causes impairment in spatial memory in the object displacement task after the end of stress. (a) The location of the four objects in the four quadrants. The displaced object was located in the quadrant C. **(b)** Representative heat plots depicting the change in exploration rate of each quadrant.**(c)**The change in exploration rate of each quadrant for the unstressed (N=10) and stressed (N=9) group (mean ± SEM). The yellow bars represent quadrant C (with the displaced object). The unstressed group shows a significant increase in exploration rate of the quadrant with the displaced object, i.e. quadrant C as compared to the other quadrants (Factor objects**:** $F_{(3, 27)}$= 2.986**,** p = 0.0486; post hoc Fischer's LSD test). The stressed group does not show any significant difference in the change of exploration for all the four quadrants (Factor objects: $F_{(3, 24)}$ = 0.269, p = 0.8474; post hoc Fischer's LSD test).An asterisk indicates significant differences (*p< 0.05 level, Fischer's LSD test for multiple comparisons).

Supplementary Figure 3

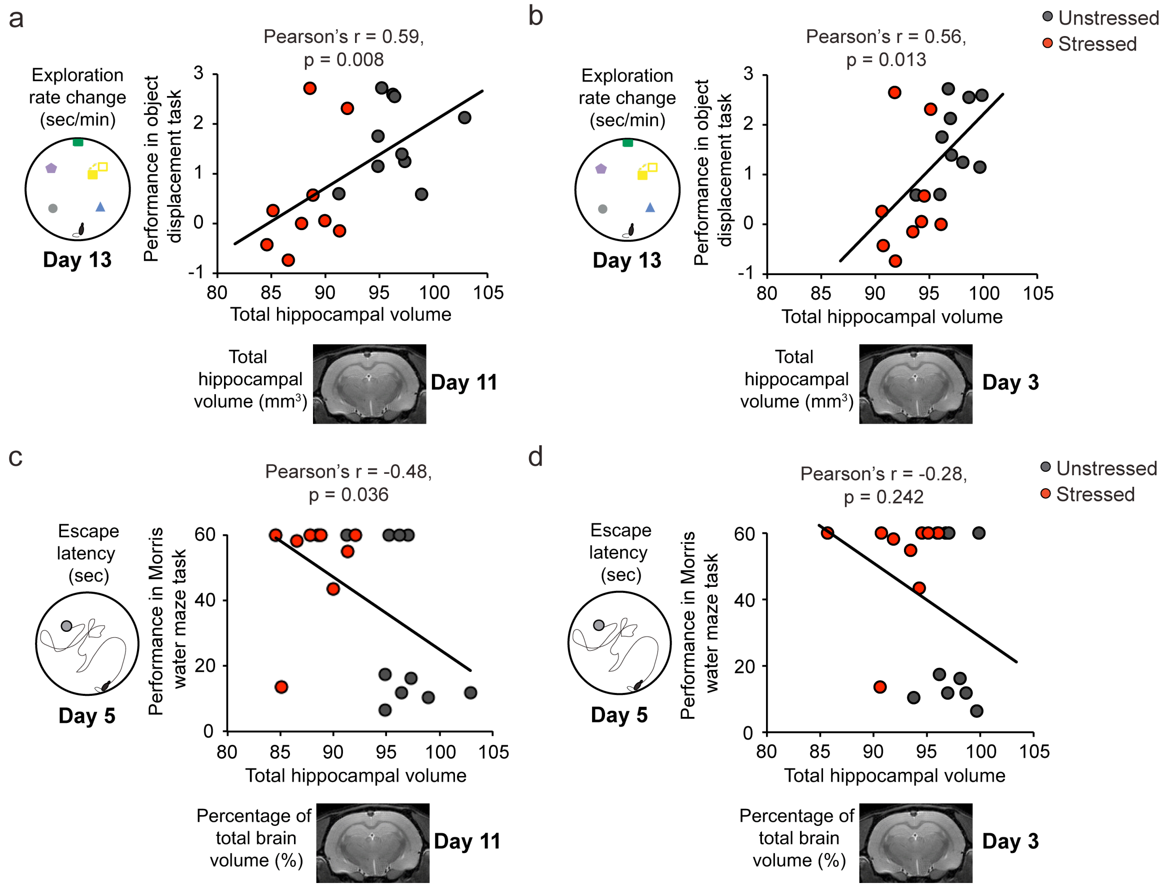

Correlation between hippocampal volume and the performance in the spatial memory tasks (a-d) Scatter plot representing individual animals. Each point represents the total hippocampal for each individual animal plotted along x-axis and the change exploration rate for each individual (a-b) or the escape latency for each individual animal (c-d) plotted along y-axis. (a) There is a significant correlation between the total hippocampal volume on day11 and the performance in the object displacement task (ODT) on day13 (Pearson's r = 0.59, p=0.008). (b) The total hippocampal volume on day3 also correlates significantly with the performance in object displacement task (ODT) on day13 (Pearson's r = 0.056, p=0.013). (c) There is a significant correlation between the hippocampal on day11 and the performance in Morris water maze task (MWM) on day5 (Pearson's r = -0.48, p=0.036). (d) There is no significant correlation between the hippocampal volume on day3 and the performance in Morris water maze task (MWM) on day5 (Pearson's r = -0.28, p=0.242).
